# Report from the Second International Symposium on Animal Genomics for Animal Health: Critical Needs, Challenges and Potential Solutions

**DOI:** 10.1186/1753-6561-5-S4-S1

**Published:** 2011-06-03

**Authors:** Steve C Bishop, Joan K Lunney, Marie-Hélène Pinard-van der Laan, Cyril G Gay

**Affiliations:** 1 The Roslin Institute and R(D)SVS, University of Edinburgh, Roslin, Midlothian, EH25 9RG, Scotland, UK; 2 Animal Parasitic Diseases Laboratory, ARS, USDA, Beltsville, MD 20705, USA; 3 INRA/AgroParisTech, UMR1313 Génétique Animale et Biologie Intégrative, F-78352 Jouy-en-Josas Cedex, France; 4 Office of National Programs, ARS, REE, USDA, 5601 Sunnyside Ave, Beltsville, MD 20705, USA

## Abstract

The second International Symposium on Animal Genomics for Animal Health held in Paris, France 31 May-2 June, 2010, assembled more than 140 participants representing research organizations from 40 countries. The symposium included a roundtable discussion on critical needs, challenges and opportunities, and a forward look at the potential applications of animal genomics in animal health research. The aim of the roundtable discussion was to foster a dialogue between scientists working at the cutting edge of animal genomics research and animal health scientists. Importantly, stakeholders were included to provide input on priorities and the potential value of animal genomics to the animal health community. In an effort to facilitate the roundtable discussion, the organizers identified four priority areas to advance the use of genome-enabled technologies in animal health research. Contributions were obtained through open discussions and a questionnaire distributed at the start of the symposium. This report provides the outcome of the roundtable discussion for each of the four priority areas. For each priority, problems are identified, including potential solutions and recommendations. This report captures key points made by symposium participants during the roundtable discussion and serves as a roadmap to steer future research priorities in animal genomics research.

## Background

One of the primary aims of the second International Symposium on Animal Genomics for Animal Health (AGAH) was to explore advances made since the first symposium [1] and to update the recommendations made in 2007 for integrating genome-enabled technologies in animal health research [2]. To achieve this aim, a roundtable discussion of leading experts in the field of genomics and animal health was organized. With contributions from the symposium participants through open discussions and a questionnaire distributed at the start of the symposium, four priorities were identified that are likely to have the greatest impact on animal health research. This report captures the problems to be solved within the four priority areas and provides potential solutions and recommendations.

1. Marker-assisted Selection of Animals with Desirable Health Traits

2. Functional Genomics of Host-Pathogen Interactions

3. Translating Genomic Information to Tools for Controlling Diseases

4. Integrating Stakeholder Support to Advance Animal Genomics in Animal Health

## Marker-assisted selection of animals with desirable health traits

Genetic studies which aim to quantify between-host variation in health-related traits or are intended to identify genetic markers associated with health have the potential to impact on many areas of disease control. However, it is only in recent years that these opportunities are beginning to be recognized by both the veterinary and animal genetics communities. Undoubtedly, it is the advent of sophisticated genomic technologies, including the availability of dense single nucleotide polymorphism (SNP) arrays in every major livestock species coupled with the prospect of resequencing large chunks of animal genomes, which have focused the attention of diverse research communities on the opportunities at hand. However, other factors have also come into play. These include notable success in the human genetics community in mapping genes controlling variation in complex diseases, the maturing of several large quantitative genetic studies investigating resistance to infectious diseases in livestock populations, and the demonstration that even field data beset by considerable noise and imprecision can still contain valuable information on host genetic variation. However, it remains true that quantitative genetic studies to identify heritable health traits and genetic marker associations are difficult and expensive. Phenotype acquisition has become the major bottleneck, with genomic information now no longer being the rate limiting step.

One of the outcomes of the AGAH symposium is the agreement that there are no generic solutions for solving the problems associated with quantitative genetic and genetic marker studies. The solutions depend upon the biology of the host animal, which generally determines the structure of the industry. Further, the industry structure influences both the nature of the predominant health problems encountered and the ease of data capture. The rapid and sustained development of genome-enabled technologies of the last few years, especially genotyping and sequencing, has provided extraordinary opportunities for advancing this field of research, however other issues remain. Participants overwhelmingly agreed that a key challenge remains the difficulty in acquiring and validating animal phenotypes. Critical needs include standardized protocols for collecting tissues and phenotypes for disease resistance traits in both experimental resource and relevant commercial animal populations. Additionally, a need was seen and agreed for proof-of-concept studies for marker-assisted selection (for disease resistance or improved health) in prioritized species. This was seen as being enhanced by multidisciplinary studies. Lastly, user-friendly and cost-effective delivery of marker-assisted selection was raised as an issue, particularly in developing countries.

### Problems to be solved

1. Availability of appropriate and reliable phenotypes, including health records

a. *Problem*: The rate limiting step in studies aiming to detect genetic markers for health is invariably the collection of phenotypes, rather than genotypes. This is a general problem in many genomic studies, including studies on non-disease traits, and is known generically as the ‘phenotype gap’. However, the situation is exacerbated for disease traits as phenotypes of disease susceptibility/resistance to infectious diseases are often more difficult and/or expensive to measure than those for performance traits. Their measurement usually requires the involvement of infection, either deliberate or through clinical history of naturally infected animals.

Various scenarios may be envisaged, and the data collection strategies will vary according to the scenario. For example, with many endemic diseases, animals may face natural challenge under field conditions. In contrast, with epidemic diseases, data collection opportunities from disease outbreaks may only be sporadic, otherwise deliberate challenge experiments may be needed. Further, some diseases are multifactorial. For example, mastitis may be caused by many pathogens, and the pathogenesis resulting from these specific infections may differ dramatically. Therefore, an important issue is whether it is possible to have generic solutions to multi-factorial diseases or, conversely, pathogen-specific solutions are required – invoking considerable extract complexity in data recording.

In many cases, disease outcomes may be the result of infection coinciding with stress induced immune suppression in livestock. An example is the respiratory diseases of livestock and poultry in intensive animal production systems caused by several different pathogens (viral and/or bacterial) in association with transport, co-mingling of pooled animals, dietary changes and administration of various veterinary treatments. Mastitis is another example of a disease where clinical cases often have an underlying issue of immune suppression in early lactation. In such cases, addressing animals’ ability to cope with stress may be as effective as concentrating on animals’ resistance to infection.

Related to the issue of stress is that of robustness. Of particular interest to breeding companies is the ability to breed animals that are robust across a variety of environments and infectious challenge conditions. This is particularly important when there are few pathogens or diseases that can be identified as overwhelming candidates for marker-assisted selection, yet general health issues are perceived. Many approaches have been advocated for addressing this issue, including interrogation of detailed health records, using animal performance as a proxy for health, and attempting to identify underlying immunological correlates of robustness.

b. *Potential solutions*: Solutions and recommendations invariably involve large scale, multidisciplinary, sometimes multi-national, projects which ‘think big’, i.e. maximize the use of animal resources and human expertise. In particular, it is critical that dialogue is established or strengthened between contributors with complementary expertise in animal health, animal genetics/genomics and other relevant disciplines.

Researchers should also be aware that opportunities to record phenotypes may occur in populations that are not standard commercial populations or well-characterized experimental lines. For example, livestock exposed to extremes of environment or disease, especially in developing countries, may be a rich reservoir of traits and gene variants related to health issues.

The goal should be to move toward more “proactive” sample collection, with specific recommendations on optimum timing and handling of blood (or other tissue) sample collection for a wide array of immunological and other disease-related studies. The now widespread availability of high-throughput technologies, including dense SNP chips for nearly all livestock species, is particularly advantageous. They allow creation of animal pedigrees, i.e. accurate estimation of genetic relatedness between individuals, and they enable quick and efficient genome scans for markers in LD with causative mutations. However, care needs to be taken with population genetic structure – mixed breed populations are likely to lead to spurious results unless sample sizes are very large and very dense SNP chips are available.

For predictable widespread diseases, international agreement is required for measurement protocols for predictable diseases. Extensive data requires that they be collected and stored on accessible databases. For emerging diseases, dialogue with governmental and other animal health policy makers to ensure timely capture of epidemic outcome data, for use in population-level quantitative genetic or epidemiological analyses. Including data collected on health traits in animal disease studies conducted in controlled laboratory settings, where the experiments are sufficiently large to provide meaningful data, will reduce the complication of uncontrolled environmental factors.

For general health issues, further research into the best means of describing host genetic effects is required. Support research to define heath traits at the molecular level. Narrowly defined phenotypes can significantly increase the power of detecting genetic factors involved in disease susceptibility and disease resistance, however they make phenotype collection more challenging. Research into the identification of animals with superior ability to withstand the immunosuppressive effects of stress may open new opportunities.

2. Development and availability of genome-associated research tools

a. *Problem*: Dense SNP chips, usually ca. 50k SNPs, are now available for nearly all livestock species. Whilst this is a luxury almost undreamt of a few years ago, these tools remain somewhat paradoxical. They are still considered too sparse for fine mapping of causal mutations, or for studies that extend across breeds or comprise genetically sub-structured populations, yet they are still expensive for routine use. SNP chips with several hundreds of thousands of markers will be more flexible, albeit expensive. However, even these are likely to have some limitations, for example incomplete LD with causative mutations at low frequencies (i.e. <0.1); it is mutations at low frequencies which may well have larger effects on the phenotype.

A significant issue with animal genome sequences is the lack of annotation, which is mostly based on homology to other genes and not actual experimental testing in the species of interest.

b. *Potential solution*: Dense SNP chips will be required for all species. However, in parallel, sparse SNP chips to be used for imputation of haplotypes will be required to reduce the cost of utilization of SNP chips for the purpose of genome-wide selection.

The big challenge, and the big opportunity, now lies in obtaining and interpreting sequence data for populations of animals. Individual genome sequences offer unparalleled opportunities for identification of causative mutations, for the generation of SNPs to be used in population-wide studies, for identification of animals with superior genotypes and for gaining additional understanding of the genetic control of traits of interest.

The same high throughput technologies that enable large scale genotyping and sequencing will also enable great insight into transcriptomics, through sequencing of transcripts. This, along with technologies that measure protein (protein arrays or mass spec) and metabolites will allow greater insight and contribute to genome annotation.

Continued efforts in genome sequencing and SNP discovery are needed. Formation of large international consortia is required to reduce per-unit cost of using SNP chips. Both high density arrays, for mapping of causal mutations, and low density arrays for haplotype imputation are required. There is also a need to support other high throughput technologies such as DNA chips and protein arrays.

3. Refining quantitative population genetic studies to usable markers

a. *Problem*: The step from discovery of significant marker associations to utilizable markers for animal health can be long, difficult and expensive.

b. *Potential solutions*: The solutions depend upon the nature of the phenotype and the architecture of host genetic resistance, i.e. whether the genetic variation is due to a small number of polymorphisms with large effects, or whether it tends to be under polygenic control.

Where phenotypes are difficult to capture, or few/single genes dominate host genetic variation, a usable marker(s) must be found. A SNP-based genome scan alone is unlikely to be sufficient, and information must be used from other sources (‘screening filters’), including comparative genomics, functional genomic studies and verification in independent animals. Once a region on the genome is found, one should (1) refine the position using more markers, (2) confirm the association in independent animals, and (3) when possible, sequence as many animals as possible for novel genetic variants.

Where genetic control is polygenic, and phenotypes are readily obtained on large numbers of animals, it is not necessary or even useful to resolve the genetic marker to a causative mutation or tightly linked marker – in this case ‘genome-wide selection’ can be used. The sum of all SNP effects across the genome can be used to predict next generation performance/resistance/health status. However, in this situation costs that are incurred on a routine basis are likely to be high, thus strategic use of sparse SNP chips for imputing haplotypes is likely to substantially reduce costs.

### Recommendations

• Support research to define health traits at the molecular level with the aim of detecting genetic factors involved in disease susceptibility and disease resistance

• Support initiatives at every level (national, regional, private, research project) that aim to proactively record defined health traits and collect samples from either commercial populations, well-characterized experimental animal lines, or where livestock and poultry are exposed to extremes of environment or epizootics

• Support the development of inter-operable databases for large-scale storage of pedigree information, phenotypes, and associated environmental factors

• Support national and international efforts that aim to sequence animal genomes, develop genome-enabled technologies, and enable their cost-effective use in research

• Support the building and integration of bioinformatics infrastructures in animal health research institutions, including the development of statistical models and software for animal genomics research

• Support research programs that focus on the the selection of animals with superior resistance to the immunosuppressive effects of stress

• Support large-scale coordinated research programs aimed at elucidating relevant aspects of host-pathogen interactions for high priority diseases

• Support meetings and workshops to enhance communication and facilitate collaborations between quantitative geneticists and experts in animal health

## Functional genomics of host-pathogen interactions

New research strategies employing high-throughput gene expression analysis are providing novel platforms for more comprehensive understanding of host–pathogen interactions. In particular, functional genomics is rapidly revolutionizing the analysis of whole genome responses of the host to environmental, nutritional, or disease factors that affect the health of animals. The symposium included several presentations on gene expression and functional genomics studies that had the aim of looking for genes that were differentially expressed. The symposium participants overwhelmingly expressed the importance of basic research and the inclusion of genome-enabled technologies in animal health research. Many of the questions and comments from the audience pointed out that the causative polymorphisms may reside on completely different chromosomes from the genes that are differentially expressed and the importance of considering epigenetic effects.

### Problems to be solved

1. Availability of validated animal disease models

a. *Problem*: There is a critical need to develop animal disease models that mimic field infections using statistically significant number of animals per group and standardized challenge inoculums. Also critical is the need to share challenge inoculums from a single source to reduce experimental variations.

There is also a major need to get better sampling tools and methods to handle and store them for multiple protein and nucleotide analyses; e.g., protocols for handling blood, saliva and oral fluids, nasal, vaginal and other easily accessible mucosal samples, urine and fecal samples.

b. *Potential solutions*: Define validated disease challenge models with respect to challenge dose, challenge inoculum that are similar to the field situation with respect to virulence and pathogen genomic sequence, and designate resource centers specialized in given disease models to share information through public websites.

2. Animals with defined disease susceptible/disease resistant phenotypes

a. *Problem*: There is a lack of livestock and poultry breeds with well characterized genotypes and disease phenotypes whose variations can be correlated with innate and acquired immune status of the host.

b. *Potential solutions*:

• Identify disease phenotypes important to industry and determine how host and pathogen genetic polymorphisms such as SNPs influence the outcome of host-pathogen interactions and disease resistance phenotypes.

• Increase the genetic analysis of animals with characterized animal phenotypes in challenge experiments to build SNP maps that define the influence of genetic polymorphisms on host immune response to pathogens.

3. Bioinformatics tools

a. *Problem*: Animal health research laboratories lack bioinformatics infrastructure: computer hardware, software, and trained scientists in computational biology. There is also a lack of animal and pathogen species-specific genomics and proteomic tools with well-annotated genome databases to predict key immunogenic epitopes in the context of different target species to understand the triggering mechanisms of the protective immune response and to evaluate vaccine-induced immunity for the animal health research community.

b. *Potential solutions*:

• Train animal health scientists in computational biology

• Build bioinformatics infrastructure within animal health research centers

• Build international animal genome databases

• Utilize available genomics resources developed in mouse and human systems, and build comparison maps for immune system genes in the target animal species

• Align international consortia with the aim of building animal genome databases as well as genome databases for important classes of animal pathogens.

4. Identification of genes that are involved in the activation/repression of key regulatory pathways

a. *Problem*: There is a need to identify molecular pathways of host and pathogens that are involved in transmission, survival, virulence, innate and adaptive immune responses, and protective immune response to natural infection and vaccination. The ultimate goal is the need to identify the “operational limits” in the genomic “imbalance” associated with current genetic selection. Basic studies are needed to enhance our understanding of the complexities of gene regulation and expression pathways, determining how species and individual genetic variations influence essential gene expression pathway and alter protein interactions. It is essential to identify where anti–pathogen responses diverge to identify pathogen specific targets.

b. *Potential solutions*:

• We need a more comprehensive approach for using functional genomics to lead us to polymorphisms that will be useful for predicting genetic merit for animal health traits. One potential approach is to use QTL studies to identify small sets of animals that are expected to differ only in whether they have favorable or unfavorable genotypes at the QTL. Use highly parallel gene expression technology (RNA-seq, microarrays or SAGE) to determine differences in expression in relevant tissue samples that are due to the QTL. Develop RT-PCR or other similar assays for the transcripts that are most highly related to QTL genotype in the small sample. Run these RT-PCR assays on the entire QTL population and redo the QTL scan. The expression data are expected to be much higher in heritability, and therefore, a much better data set for QTL detection, than direct disease data. This QTL scan should provide much better information for locating the position of the QTL and establishing relationships between haplotypes and disease resistance that should hold up across multiple breeds.

• Develop genome-wide high throughput transcriptional profiling systems to study host-pathogen interactions and identify key candidate host and microbial genes that are associated with infection, pathogenicity, antigenic variation and host range specificity.

• Invest more resources and time into the interactomics and metabolomics area.

5. Development and availability of reagents to study gene function

a. *Problem*: There is a need to develop a full set of specific research reagents for the key gene products involved in immune functions. Pathways identified for human and mouse species may not be applicable to food animal species. There is a need to develop species specific information. This requires basic set of species specific reagents, databases to track homologies and differences.

b. *Potential solutions*:

• Identify research centers with capability to develop immune reagents and specific knock-out tools (e.g., siRNAs).

• Expand existing research projects on the development of immunological reagents for different animal species.

6. Discovery of biomarkers

a. *Problem*: It is paramount that the animal health research community has access to measurable biomarkers linked to disease susceptibility, disease resistance, and mechanisms of protective immunity. There is still a major need to get better sampling tools and methods to handle and store samples for multiple analyses, e.g., transcriptome, proteome, metabolome, and protocols for handling blood, saliva and oral fluids, nasal, vaginal and other easily accessible mucosal samples, urine and fecal samples. There is also a need to identify biomarkers for healthy animals, whether that be better responders to pathogens or to vaccinations. Final outcome could be an animal that responds more favorably with its innate immune response. However, it is important that selection for “robust animal” excludes the selection for non-desirable traits. Important factors for each species must be identified so we able to balance tradeoffs during the selection process.

b. *Potential solutions*:

• Conduct biomarker discovery studies with “standardized” validated animal models that have well defined phenotypes.

• Increase the use of proteomics and transcritomic technologies in biomarker discovery studies and implement the rational use of proteomics data to develop specific biochips/protein chips for genomics research.

• Identify techniques to effectively assess phenotype at multiple stages of host –pathogen interaction: infectious course, disease pathology, recovery and persistence. These must be robust to address current commercial stock as well as genetics relevant to small farms and developing economies.

### Recommendations

• Support the development of experimental animal challenge models that provide measurable phenotypes relevant to actual diseases under field conditions

• Support long-term projects that will build the infrastructure needed to conduct the extensive phenotyping and genotyping of animal populations relevant to animal health priorities

• Support research programs that aim to identify genetic markers that correlate with disease phenotypes, and carry out whole genome expression analyses using tissue-specific microarrays and RNA-seq to identify genome sequences that influence disease parameters

• Support functional genomics research programs that aim to identify genetic markers that are linked to regulatory pathways that modulate host-pathogen interactions

• Support research programs in relevant animal hosts that aim to confirm the association of genetic markers with host responses in large animal populations

• Support the establishment of core bioinformatics infrastructures with state-of-the-art genomic tools and resources that can integrate genotype, phenotype and functional genomics data for agricultural animal species and their respective priority diseases

• Support genome database initiatives for priority animal species and pathogens

• Develop proteomics tools to support animal health research projects that have the aim of identifying host molecules and biomarkers that are associated with or have a role in modulating host-pathogen interactions

• Establish an international consortium to advance and enable the development of reagents to study gene regulation, gene function, and disease pathways in priority animal species

• Support initiatives to integrate genetic, functional genomics, and bioinformatics data in a systems biology approach to understand complex diseases

• Organize & manage all interactome research efforts using the technology readiness level standard for improving the efficiency of moving from concept to reduction to practice for direct application in the animal production systems.

• Create, test & validate comprehensive predictive biology models for optimizing animal health in the prioritized pathogens in the prioritized livestock species.

## Translating genomic information to tools for controlling diseases

Genomic tools are providing new opportunities for understanding genetic diversity, the genetic variation associated with diseases processes, and host-pathogen interactions. The scientific information generated from the use of these tools in animal health research will enable the rational design of highly effective diagnostics, vaccines, drugs, biotherapeutics, and animals resistant to diseases.

### Problems to be solved

1. Using animal genomes to understand genetic diversity and inform best management practices to improve animal health and animal production systems.

a. *Problem*: There is a critical need to preserve animal genetic diversity and to understand the role of genetic variations on host responses to diseases, drug treatments, and vaccination.

b. *Potential solutions*:

• Use genome wide genetic markers and novel methods to actively manage animal biodiversity

• Study the dynamics of specific and general response to disease challenges, especially innate immunity.

• Evaluate animal disease models that try and replicate “real world” disease complexes, such as the polymicrobial infections associated with the bovine respiratory disease complex.

• Establish general guidelines for using genomic tools in drug and vaccine clinical trials to identify the genotype of study animals and their response to the challenge and/or test treatment.

2. Easy to use diagnostic platforms for genetic tests, forensic veterinary medicine and traceability.

a. *Problem*: There is a lack of rapid, robust, accurate and cost effective genetic tests to use in analyzing disease outbreaks for both the disease agent and the host.

b. *Potential solutions*:

• Develop molecular diagnostics for genotyping hosts and pathogens

• Develop diagnostic platforms to predict new and emerging high-virulence pathogenic strains and the expected susceptible animal populations

• Develop molecular diagnostics to facilitate the traceability of animals with desired or undesired health traits

3. Selection of animals with disease susceptible and resistant traits

a. *Problem*: There is a need to integrate studies on disease resistance with selection programs. This will require strategic collaborations with commercial breeding companies.

b. *Potential solutions*:

• Establish collaborations from an early stage using animals in well structured populations with good records and already well characterized phenotypes.

• Animal challenge models must be commercially realistic and include polymicrobial /combined disease challenges.

• Establish correlated responses to selection for any marker.

4. Application of genome-enabled technologies in drug and vaccine discovery, including transgenic animals that are resistant to disease and the selection of animals that respond effectively to drugs and vaccines.

a. *Problem*: The application of animal genome-enabled tools will demand fundamental changes in the approaches used to discover and develop animal drugs and vaccines, including their delivery and application in disease control programs. One of the main challenges is the total absence of regulatory guidelines for the applications of these new technologies, including transgenic animals. The ability to design drugs and vaccines for genetically defined animal populations will require additional planning. For instance, in the area of vaccines, there is lack of established “vaccine profiles” to identify specifically what a vaccine is expected to achieve (e.g., efficacy with one dose, mass vaccination capability, prevent disease transmission, etc) for specific diseases in individual animals and targeted commercial populations. Vaccines must not only be designed to achieve the desired vaccine profile but must also anticipate the genetic variations of the host and pathogens to enable predicted outcomes in vaccination campaigns and the selection of good responders to vaccination.

b. *Potential solutions*:

• Support vaccine discovery research programs that target vaccines specifically designed for control and eradication.

• Support vaccine studies that assess new pathogenic strains, especially ‘escapes’ from vaccine protection.

• Integrate genomic tools in drug and vaccine studies to assess the effects of genetic variation on efficacy and the control of disease in defined animal populations.

• Support vaccine studies that take into account age dependent effects of vaccines and adjuvants with maturation of the immune system.

• Support research programs that integrate genomic and transgenic technologies to achieve disease resistance

• Include regulatory agencies in the early phases of research programs that integrate genome-enabled tools in study designs

• Establish strategic research collaborations with commercial breeding companies and pharmaceutical companies.

### Recommendations

• Support initiatives that will use animal genomic innovations to assess and manage domestic and wildlife animal genetic diversity

• Support studies that will lead to the development of rapid, accurate and cost-effective molecular genetic tests for host and pathogens to analyze field disease outbreaks and to develop predictive diagnostics

• Integrate disease resistance traits with existing multi-trait selection programs

• Integrate genetic profiling in the development of disease models for drug and vaccine discovery research

• Support initiatives that integrate genomic approaches to vaccine development, including genetic and immune variations in target animal populations

• Support transgenic animal research programs that target disease resistance

• Support research programs that include collaborations between animal health research institutions, commercial breeding companies, and pharmaceutical companies

## Integrating stakeholder support to advance animal genomics in animal health

The promise of animal genomics will not be achieved without stakeholder support. The burden of communicating the significant opportunities that genome-enabled technologies offer for improving animal health lies squarely on the shoulders of the animal health research community.

### Problems to be solved

1. Collaborations between the public and private sectors

a. *Problem*: Animal health is a very broad area with different diseases affecting different sectors, species, and regions. There is a lack of understanding of the opportunities for new approaches and in particular what is required for progress. There is a critical dearth of funding for animal health research and bias against non-hypothesis led approaches.

b. *Potential solutions*:

• Establish priorities for research – specific diseases, general “robustness.”

• Establish funding mechanisms that enable researchers to work with producers and provide access to large numbers of relevant animals.

• Plan to exploit disease problems at the commercial level including “SWAT teams” to collect phenotypic information and appropriate samples (including environmental data).

2. Intellectual property and technology transfer to industry

a. *Problem*: Too many groups involved in “protecting” IP before any is developed, inhibiting cooperation or blocking progress. Adversarial approaches to protection predominate. Suspicion of need for profitable application. Possible competition between genetic and “pharma” approaches reducing investment.

b. *Potential solutions*: Seek to develop approaches based on pre-competitive research.

Seek an “open” shared approach to generating “next generation” IP. Create “incubator” opportunities to take results to application.

3. Impact on veterinary education and animal and poultry science

a. *Problem*: Little dialogue between veterinarians and animal and poultry science particularly in relation to the genetics of animal health. The focus tends to be on disease challenge or *in vitro* approaches with limited numbers of animals or genetic variation.

b. *Potential solutions*:

• Seek to stimulate multidisciplinary teaching across schools.

• Seek to stimulate joint research across disciplines.

• Seek to stimulate “host-centered” approaches.

4. Contributions of veterinary practitioners, animal health professionals, and animal agriculture allied industries

a. *Problem*: There is a lack of a unified vision on the opportunity of genomics for animal health; a lack of understanding of actions required to investigate the genetics of animal health; and poor definition of new tools required to undertake the required research.

b. *Potential solutions*:

• Develop a strategic vision for animal genomics for animal health setting out the roles of each group of industry professionals.

• Create a communication program to stimulate interest of, and dialogue between, these groups.

• Focus on solutions that work at the population level.

5. Support from regulatory agencies to enable these new technologies

a. *Problem*: Regulatory environment for “gene-based” technologies, e.g. Genetic Modification, Cloning, RNA interference, DNA vaccination, is unclear and inhibits research (as well as commercialization).

b. *Potential solutions*:

• Develop science-based approach to “technical” aspects of regulation.

• Utilize “participatory” approaches to stimulating public debate and informing the regulatory process.

• Develop a regulatory system to deal with issues of public acceptability in parallel with the technical aspects.

• Benchmark other industries in relation to “licensing” of new and/or advanced technology.

### Recommendations

• Support the funding of innovations in animal genomics research specifically designed to solve problems in animal health

• Develop integrated approaches that include the development of sample repositories and databases that support research networks

• Develop more “epidemiological” approaches to the genetics of animal health and integrate in veterinary and animal science educational programs

• Establish and support demonstration projects to deliver concrete examples of the value of integrating genome-enabled technologies in animal health research programs

• Establish a progressive international regulatory framework that is science-based and provides a clear path for registering animal disease control tools derived from genome-enabled technologies

• Support meetings to align veterinarians, animal scientists, and genomics/genetics experts to foster a dialogue and educate on the importance of multidisciplinary approaches addressing the application of genome-enabled technologies to animal health

## Conclusion

The availability of new animal genomics tools provide extraordinary opportunities for solving some of the most pressing and difficult problems in animal health research. Animal genomics also provides new innovative approaches for controlling diseases affecting domestic animals. Quantitative genetics, functional genomics, translating genomics information into tools for controlling diseases, and integrating stakeholder support were identified as four priority areas for animal health research. Investing in basic research was also identified as critical for the sustainability and success of animal genomics research in animal health. The application of new genome-enabled technologies in animal health research has the potential of significantly improving the health and welfare of animals, resulting in improved efficiency, sustainability, biosecurity, and the protection of animals worldwide. This report provides a roadmap for moving forward by providing recommendations and concrete steps to achieve the impact that animal genomics offers animal health research.

## List of abbreviations used

GWAS: genome-wide association studies; LD: linkage disequilibrium; QTL: quantitative trait loci; siRNA: small interfering RNA; SNP: single nuclear polymorphism.

## Competing interests

The authors declare that they have no competing interests.
